# Editorial: Treatment resistant depression (TRD): epidemiology, clinic, burden and treatment

**DOI:** 10.3389/fpsyt.2025.1588902

**Published:** 2025-03-18

**Authors:** Andrea Fiorillo, Koen Demyttenaere, Vassilis Martiadis, Giovanni Martinotti

**Affiliations:** ^1^ Department of Psychiatry, University of Campania “L. Vanvitelli”, Naples, Italy; ^2^ Faculty of Medicine, University Psychiatric Center, KU Leuven, Leuven, Belgium; ^3^ Department of Mental Health, Asl Napoli 1 Centro, Naples, Italy; ^4^ Department of Neurosciences, Imaging and Clinical Sciences, Università degli Studi G. D’Annunzio Chieti-Pescara, Chieti, Italy

**Keywords:** depression, TRD (treatment-resistant depression), burden, pharmacological treatment, epidemiology

Depressive disorders are severe mental disorders, with a lifetime prevalence of 16% in the general population, associated with a significant personal and social burden. Median age of onset, basic sociodemographic and environmental correlates, symptom profile and severity of depression are generally comparable across different countries and cultures. Depressive disorders can be episodic or recurrent, depending on clinical, personal and social variables (1, 2).

Most patients with major depression report an incomplete and inadequate clinical remission, with many residual symptoms, cognitive dysfunctions and working impairment (3, 4); up to one out of three patients do not fully respond to currently available treatments. According to the FDA and EMA, patients are considered to have treatment-resistant depression (TRD) when they fail to respond to ≥2 successive adequate trials of antidepressants in a single episode (5, 6). The terminology, definition and clinical usefulness of the concept TRD is debatable for multiple reasons (7). First, difficult-to-treat depression or (multiple) treatment failure are probably less stigmatizing terms. Second, it has been demonstrated that there are no meaningful cut-offs between patients having experienced 2, 3 or 4 consecutive failures suggesting more continuous ‘staging models’ of treatment failures. Third, we lack studies to scientifically guide clinicians on what to do after 1, 2, 3 or more treatment failures (guidelines are rather consensus based than evidence based). Despite these conceptual comments, TRD is a common condition, with a prevalence rate ranging from 30- to 40% of patients treated with antidepressants, and it is associated with high levels of personal and societal burden. Treatment-resistant depression is associated with a significant burden for patients, caregivers and families, increasing disability and worsening quality of life. Although several sociodemographic, contextual and psychological factors (e.g., living alone or together, being employed or unemployed, cognitive functioning) (8, 9), and several clinical factors (e.g., unipolar or bipolar depression, lifestyle behaviors) can influence clinical outcome in persons with depression, only a few factors are considered as predictive of non-response across multiple modalities of treatment (10–12). Therefore, there is the need to carry on further studies to investigate how to improve the personalized approach to people suffering from TRD.

In recent years, the therapeutic armamentarium of clinicians for treatment of depression has been improved by innovative pharmacological and non-pharmacological/brain stimulation therapies (ECT, TMS, VNS) (13). More recently, new pharmacological approaches focusing on psychedelic-derived drugs (e.g., ketamine, esketamine, psylocibin) have been studied, providing clinicians with new treatment choices.

Our Research Topic entitled *“Treatment Resistant Depression (TRD): epidemiology, clinic, burden and treatment*” includes more than 20 papers written by researchers and clinicians coming from different world regions. While some papers deal with the topic of diagnosis, early detection and clinical features of TRD (Pettorruso et al.; Liu and Read; Baune et al.; Mancuso et al.), the vast majority address the topic of treatment options for TRD, including brain stimulation therapies, novel pharmacological agents and new treatment-delivery modalities (Dragon et al.; Aboubakr et al.). Finally, we received and accepted some systematic reviews and metanalyses dealing with the role personality disorders in moderating the effectiveness of treatment for TRD (Więdłocha et al.), the efficacy of ketamine/esketamine for unipolar and bipolar depression (Rodolico et al.), the use of neuromodulation for treating TRD (Lan et al.), which complement research-driven data with those derived from real-world trials (Chrenek et al.; Menculini et al.; Di Vincenzo et al.; Pessina et al.).

Given the high number of submissions and of accepted papers of extremely good quality, we can definitely consider that the present Research Topic has been extremely successful. However, despite a growing interest on TRD (from its definition to the diagnosis and to treatment options), information collected cannot be considered as conclusive yet, but can represent the basis for future studies. We are extremely grateful to all researchers, patients and caregivers that have participated in these studies, and we are committed to further increase the knowledge in the field.
